# Protocol for the development of a bioluminescent AML-PDX mouse model for the evaluation of CAR T cell therapy

**DOI:** 10.1016/j.xpro.2024.103522

**Published:** 2024-12-12

**Authors:** Mireia Mayoral Safont, Calum Leitch, Mihaela Popa, May Eriksen Gjerstad, Benjamin Caulier, Else Marit Inderberg, Sébastien Wälchli, Pascal Gelebart, Emmet Mc Cormack

**Affiliations:** 1 Precision Oncology Research Group, University of Bergen, Bergen, Norway; 2 Department of Clinical Science, University of Bergen, Bergen, Norway; 3 Department of Hematology, Haukeland University Hospital, Bergen, Norway; 4 Centre for Pharmacy, Department of Clinical Science, University of Bergen, Bergen, Norway; 5 Centre for Cancer Biomarkers (CCBIO), University of Bergen, Bergen, Norway; 6 Translational Research Unit, Section for Cellular Therapy, Department of Oncology, Oslo University Hospital, Oslo, Norway; 7 Kinn Therapeutics, Bergen, Norway

**Keywords:** cell biology, cell culture, flow cytometry, cancer, immunology, model organisms, molecular biology

## Abstract

Patient-derived xenograft (PDX) models of acute myeloid leukemia (AML-PDX) offer advantages over cell line models by capturing the complexity and heterogeneity of patient-derived samples. Here, we present a protocol for developing a bioluminescent AML-PDX model in mice to evaluate chimeric antigen receptor (CAR) T cell therapy. We describe steps for transducing, engrafting, expanding, and enriching AML-PDX cells. We then detail procedures for *in vitro* and *in vivo* validation of the AML-PDX model for the evaluation of CAR T cell immunotherapy.

For complete details on the use and execution of this protocol, please refer to Caulier et al.^1^

## Highlights

• Steps for transduction of AML-PDX cells using Red F-luc-GFP lentiviral particles
• Guidance on engraftment of transduced AML cells into immunodeficient mice
• Instructions for the enrichment of AML cells by flow cytometry and their repassage in mice
• Procedure for AML-PDX^LUC^ model validation and CAR T efficacy evaluation

## Abstract (editor highlights)

Publisher’s note: Undertaking any experimental protocol requires adherence to local institutional guidelines for laboratory safety and ethics.

## Graphical abstract

**Figure:**
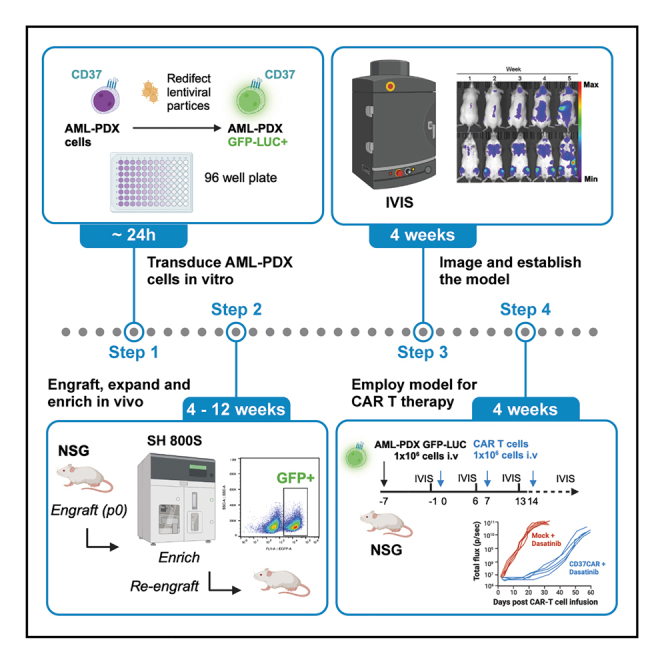

## Before you begin

This protocol outlines the development of a bioluminescent AML PDX model for the evaluation of CAR-T therapy, utilizing established and well-characterized PDX models engrafted in immunodeficient mice. The AML-PDX samples isolated from the spleen of engrafted mice are frozen in 90% FBS and 10% DMSO and stored in liquid nitrogen. The example provided in this protocol uses an AML-PDX model predetermined to express CD37 as this was the target antigen identified for CAR T cell therapy. However, the model generated can also be employed to evaluate other immunotherapeutic and small-molecule drugs. When a specific target antigen is needed for therapeutic efficacy, pre-screening of PDX material should be performed to confirm the presence of the target antigen. NSG mice were used for the protocol outlined, though other immunodeficient strains may also be suitable. Specific-pathogen-free conditions for animal housing are required and the mice should be acclimatized for one week before the study starts. Cell transductions should be conducted in a Biosafety Level 2 (BSL-2) laboratory. Prior experience in handling laboratory mice and proficiency in analyzing flow cytometry data are necessary.

### Institutional permissions

All animal experiments require approval from the local Ethical Committee. The procedures described here were performed following the Norwegian Commission for Laboratory Animals and approved by the Norwegian Food and Safety Authority. The patient samples used to generate the PDX models were obtained following approval from the Norwegian Regional Committee for Medical and Health Research Ethics (REK) and with written informed consent from the patient.

## Key resources table

**Table:** 

REAGENT or RESOURCE	SOURCE	IDENTIFIER
**Bacterial and virus strains**		

IVISbrite Red F-luc-GFP lentiviral particles	Revvity Inc.	CLS960003

**Biological samples**		

Cryopreserved PDX cells from mouse spleen	University of Bergen	N/A
Human: CD37CAR T cells	University of Oslo	N/A

**Chemicals, peptides, and recombinant proteins**		

RPMI-1640	Sigma-Aldrich	Cat# R5886
L-glutamine solution (200 mM)	Sigma-Aldrich	Cat# G7513
Penicillin-streptomycin (100×)	Sigma-Aldrich	Cat# P0781
Trypan blue stain	Invitrogen	Cat# T10282
Hexadimethrine bromide (polybrene)	Sigma-Aldrich	Cat# H9268
Dimethyl sulfoxide (DMSO)	Sigma-Aldrich	Cat# D2650
Ethylenediaminetetraacetic acid (EDTA)	Sigma-Aldrich	Cat# 03685
Sodium azide (NaN_3_)	Sigma-Aldrich	CAS# 26628-22-8
Fetal bovine serum (FBS)	Sigma-Aldrich	Cat# F7524
IsoFlo Vet 250 mL	Zoetis	Cat# 002185
D-luciferin firefly potassium salt	Biosynth	Cat# L-8220
Phosphate-buffered saline	Sigma-Aldrich	Cat# P4417
Sodium chloride solution 9 mg/mL	B. Braun	Cat# 363 1982
CD37-Alexa Fluor 647	Santa Cruz	Cat# sc-18881

**Experimental models: Organisms/strains**		

Mouse: NOD.Cg-Prkdcscid Il2rgtm1Wjl/SzJ (NSG) Female 6–10 weeks old	Jackson Laboratory	Cat#005557

**Software and algorithms**		

FlowJo software	FlowJo, LLC	https://www.flowjo.com/
BD FACS Diva software	BD Biosciences	https://www.bdbiosciences.com
SH800 software	Sony	https://www.sonybiotechnology.com
Living Image 4.7.3	Revvity Inc.	https://www.revvity.com
GraphPad Prism 10	GraphPad Software	https://www.graphpad.com/

**Other**		

X100 Omnican 50 Insulin syringes 0.5 mL/50	B. Braun	Cat# 9151125S
96-well plates	Sarstedt	Cat# 83.3924.500
96-well plates, black walled	Corning	Cat# 4680
6-well plates	Sarstedt	Cat# 83.3920500
50 mL centrifuge tubes	Sarstedt	Cat# 62.547.254
15 mL centrifuge tubes	Sarstedt	Cat# 62.554.502
2 mL cryogenic vials with screw cap	VWR	Cat# 479-1262
1.5 mL microcentrifuge tubes	VWR	Cat# 525-1164
10 mL serological pipettes	Sarstedt	Cat# 86.1254.001
Cell strainers 40 μm pore size	VWR	Cat# 732-2757P
Bürker counting chamber	Fisher Scientific	Cat# 10628431
Cell freezing container	Corning	Cat# CLS432001
Inverted microscope	N/A	N/A
Stainless steel forceps	AgnThos	Cat# 11651-10
Dissecting scissors	AgnThos	Cat# 14002-12
Microscope slides	VWR	Cat# 631-1554
10 μL micropipette	Eppendorf	Cat# 3123000020
100 μL micropipette	Eppendorf	Cat# 3123000047
1000 μL micropipette	Eppendorf	Cat# 3123000063
Cell culture dish 100 × 20	Thermo Scientific	Cat# 150466
FACS tubes	Sarstedt	Cat# 55.1578
IVIS Spectrum optical imager	Revvity Inc.	N/A
SH800S Cell sorter	Sony	N/A

## Materials and equipment

### Preparation of reagents

**Timing: 30 min**

**Table:** FACS buffer

Reagent	Final concentration	Amount
Fetal bovine serum	5% (v/v)	10 mL
EDTA	2 mM	117 mg
NaN_3_	0.1% (w/v)	200 mg
1× PBS	N/A	190 mL
**Total**	**N/A**	**200 mL**

Storage conditions: 4C for up to 3 months.

**Table:** Complete RPMI-1640

Reagent	Final concentration	Amount
RPMI-1640	N/A	440 mL
L-Glutamine solution (200 mM)	2 mM	5 mL
Penicillin-streptomycin (100×)	5%	5 mL
Fetal bovine serum (FBS)	10%	50 mL
**Total**	**N/A**	**500 mL**

Storage conditions: 4°C for up to 1 month.

**Table:** Transduction medium

Reagent	Final concentration	Amount
RPMI-1640 Complete	N/A	280 μL
Polybrene (1 mg/mL)	8 μg/mL	1.6 μL
RediFect Red-FLuc-GFP	MOI 3	120 μL
**Total**	**N/A**	**400 μL**

Transduction media should be freshly prepared for its use. Keep on ice while handling the cells. Values described in reflect the transduction of 4 × 10^5^ AML-PDX1 cells, with MOI 3, transduced across 4 separate wells of a 96-well plate.

**CRITICAL:** Transduction media should be prepared immediately before transduction and handled in a cell culture bench that is prepared and approved for lentivirus work.

## Step-by-step method details

### Transduction of AML-PDX cells

**Timing: 24 h (per transduced PDX model)**

Transduction of AML-PDX cells is performed *in vitro* followed by immediate re-injection into NSG mice. Successful transduction is crucial for establishing the bioluminescent model and conditions should be optimized to achieve the best possible transduction efficiency and recovery of viable cells for engraftment.

***Note:*** The optimal culture conditions (medium and cell density) may vary across PDX samples and tests should be performed to determine the best conditions for the selected model.

1. Thaw frozen AML-PDX cells (5–20 × 10^6^) rapidly in a water bath at 37°C and transfer immediately to 10–20 mL of pre-warmed RPMI containing 20% FBS in a 50 mL centrifuge tube for 5 min, 300 × *g*, RT, to achieve a concentration of cells suitable for counting (0.5–1 × 10^6^ cells/mL). Troubleshooting 1
2. Take a 10 μL aliquot from the volume and count cells using Trypan blue in a Bürker’s chamber to determine the total number of viable cells recovered.
3. Based on counts, transfer the volume of cell suspension containing the amount of cells to be transduced into a centrifuge tube and spin for 5 min, 300 × *g*, RT.

***Note:*** The volume pipetted into the tube will depend on the amount of viable patient cells recovered and the amount intended for transduction. Aim to have a minimum of 1 × 10^5^ cells/mouse available post-transduction for injection. To account for spontaneous apoptosis and the stress of the transduction protocol we suggest an initial seeding of 4 × 10^5^ cells per mouse.

**CRITICAL:** The next steps should be performed in a cell culture bench equipped to handle viral samples.

4. Thaw a vial of RediFect lentiviral particles (GFP-luciferase) on ice. Prepare the required volume of the transduction medium. Troubleshooting 2

***Note:*** In this study, a multiplicity of infection (MOI) of 3 was employed. The selected MOI should account for the number of cells needed for subsequent engraftment. A range of MOIs should be tested if the optimal MOI is unknown. A range from MOI 1 to 20 is recommended for AML-PDX cells based on the author's experience.

5. Centrifuge the tube at 300 × *g*, discard the supernatant and resuspend the pellet in the transduction medium. Keep the cell suspension at a concentration of 1 × 10^6^ cells/mL.
6. Aliquot the cell containing transduction medium into a 96-well plate by adding 100 μL to each well.

**CRITICAL:** AML-PDX cells can be transduced in a flat-bottomed, untreated 96-well plate across as many wells as required, keeping the following ratio: 1 × 10^5^ cells/100 μL/well.

**Figure 1:**
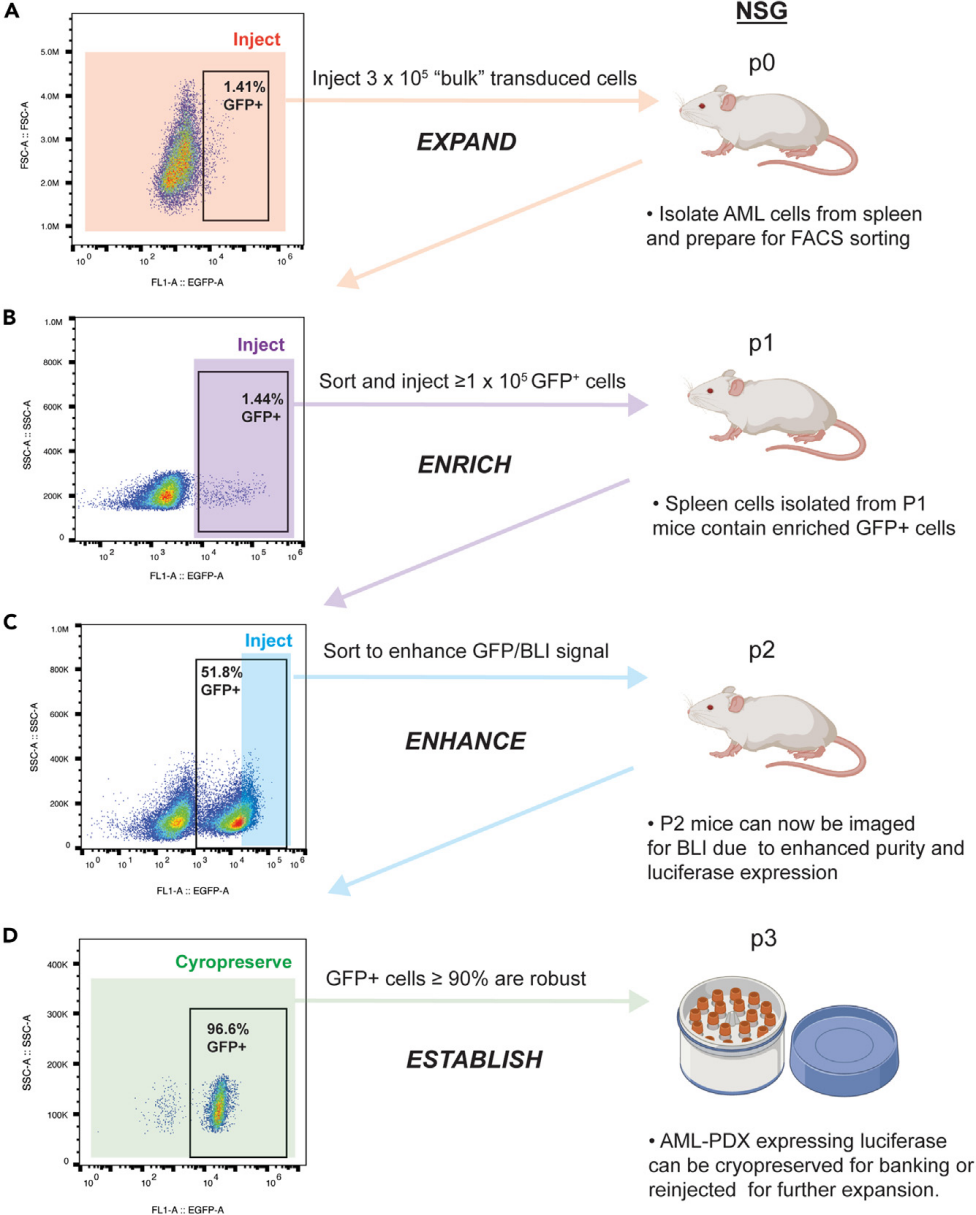
Key steps for expansion and enrichment of GFP-LUC+ AML-PDX cells via FACS and *in vivo* engraftment in NSG mice p = passage. (A) Following low transduction efficiency (1.41%), all 3 × 10^5^ AML-PDX cells exposed to lentiviral particles are injected into a recipient NSG mouse to ensure engraftment and facilitate expansion of the GFP positive population. (B) The GFP+ cell population (1.44%) remains stable in P0 mice, allowing for sorting and enrichment for further engraftment (purple). (C) 51.8% of AML-PDX cells isolated from the spleen of P1 are GFP+. The top 20% highest GFP-expressing cells are FACS sorted and injected into P2 mice (blue). (D) Spleen cells from P2 mice are 96.6% GFP+ and cryopreserved for future use.

7. Spin the 96-well plate at 32°C, 1000 × *g* for 90 min.
8. Remove the plate from the centrifuge and fill all empty wells with 300 μL of sterile saline to keep humidity and prevent evaporation.
9. Place it in an incubator at 37°C with 5% CO_2_.
10. Incubate the AML cells overnight in the transduction medium.
11. After 16–20 h of incubation with the lentiviral particles, collect the cells from all the wells in an Eppendorf tube.
12. Spin at 300 × *g* for 5 min at RT.
13. Remove supernatant into a solution containing 10% fresh bleach (or similar) and resuspend cells in sterile PBS solution and count.
14. Analyze the transduction rate by the GFP expression in a small portion of the transduced cells by flow cytometry.
  a. Pipette a minimum of 50.000 cells for flow cytometry analysis.
  b. Spin down the cell suspension at 300 × *g* for 5 min at RT.
  c. Remove supernatant.
  d. Resuspend cells in PBS keeping a concentration between 0.1 and 0.5 × 10^6^ cells/mL.
  e. Acquire cells on flow cytometer and analyze the results using the FlowJo software (Figure 1A).

***Note:*** As AML-PDX cells are typically non-proliferative *in vitro* and may exhibit low transduction efficiency, transduced cells are immediately prepared for passage in mice to expand the total number of GFP-LUC positive cells available for sorting.

### Engraftment of transduced AML cells into immunodeficient mice

**Timing: 30–90 days (depending on the disease latency of the AML-PDX before transduction)**

All transduced cells, including both GFP-positive and GFP-negative populations as indicated in Figure 1A, are injected into mice via the tail vein.

15. Prepare transduced cells for injection.
  a. Count the cells.
  b. Resuspend the cells in a small volume of sterile saline by keeping the cell density to 0.1–1 × 10^6^ cells per 100–150 μL.
  c. Fill 2–3 insulin syringes (30 gauge) with 100–150 μL cell suspension.
    ***Note:*** It is recommended to use a minimum of 0.1 × 10^6^ cells per syringe to ensure successful engraftment. The number of cells required may vary depending on the PDX model and should be determined based on previous experience with the model.
  d. Keep syringes on ice until cells are injected.
16. Inject the transduced cells into immunodeficient mice via tail-vein.

***Note:*** There are various methods for mice IV injections. In this protocol, a custom-made restrainer equipped with a light source was utilized. The light enhances the visualization and dilation of the mice’s tail vein, facilitating efficient injections.

17. Monitor engraftment of AML cells. Troubleshooting 3
  a. Weigh mice 3 times a week and record any weight loss.
  b. Observe mice for the presence of other clinical signs such as paleness, ruffled fur or hind limb paralysis.
  c. Euthanize the mice when the humane endpoints are reached.
  d. Collect the spleen in a tube containing sterile media.
18. Prepare a single-cell suspension from the spleen.
  a. Place the spleen and medium in a Petri dish.
  b. Mechanically dissociate spleen tissue using two microscope slides, using the short edges of the slides to disrupt the spleen tissue.
  c. Pass cell suspension through a 40 μm cell strainer into a 50 mL tube.
  d. Spin down cells for 5 min at 300 × *g* at RT.
  e. Resuspend the cells with 10–15 mL sterile saline.
  f. Count cells.

***Note:*** A tissue grinder or a spleen dissociation kit can be used as an alternative to the method described here. Depending on the model, we typically expect between 10 and 600 × 10^6^ AML-PDX blasts from a single spleen of a mouse with advanced disease.

19. Proceed with cell sorting of positive cells.

***Note:*** If the cells cannot be sorted right away, they can be frozen down viable in freezing media (10% DMSO and 90% FBS) and analyzed/sorted at a later time point.

### Enrichment of GFP+ cells by FACS and reinjection into immunodeficient mice

**Timing: Variable (depending on the disease latency of the AML-PDX before transduction and the number of cycles for enrichment needed)**

In this step, cells are sorted based on GFP expression and reinjected into immunodeficient mice to further expand the number of GFP-LUC+ cells.

20. Adjust the single-cell suspension from the spleen to a concentration between 5 × 10^6^ to 20 × 10^6^ cells/mL of sterile PBS for effective cell sorting.
21. Load cells into SH800 Sony cell sorter to isolate GFP-LUC-expressing cells. Sort at a flow rate of 200–1000 events per second.
22. Collect a sufficient number of GFP-LUC+ cells in complete RPMI medium (between 0.1 × 10^6^ and 1 × 10^6^ cells/mouse) for subsequent engraftments. (Figure 1B).

**CRITICAL:** The duration of the sorting process is dependent upon the percentage of GFP+ cells, as well as the desired purity and viability. It is important to avoid prolonged storage of cells in the collection tube post-sorting.

23. After collecting the cells, spin down at 300 × *g*, 5 min, RT, and remove the supernatant.
24. Resuspend in sterile saline to reinject into immunodeficient mice (as described in steps 15–16).
25. Monitor engraftment, collect, and process tissue, and enrich the positive population for GFP-LUC cell expression as required by repeating steps 18–24.

***Note:*** FACS enrichment/*in vivo* expansion may be repeated until the GFP-LUC+ percentage of cells is determined to be sufficiently bright for *in vivo* imaging. In this example, 3 passages of enrichment and expansion were required as indicated in Figure 1**.** Following passage 1, the 20% brightest was collected for expansion as indicated by blue shading in Figure 1C.

26. When cells are judged to be sufficiently bright and pure by FACS, perform a final round of injection in NSG mice as for steps 15–16.
27. Monitor the mice, harvest material, and analyze the cells by flow cytometry as in steps 17–18.
28. Analyze GFP expression by flow cytometry to confirm the purity of the established model (Figure 1D).

### *In vitro* and *in vivo* validation of AML-PDX^LUC^ model

**Timing: Variable (depending on the disease latency of the AML-PDX used)**

The quality of the transduced cells is assessed through an *in vitro* BLI assay, which quantifies the mean luminescent intensity per transduced cell. For *in vivo* model validation, disease progression and metastasis are monitored by weekly BLI imaging, establishing a timeline for these events. For the model used as an example in this protocol all bioluminescent imaging, both *in vitro* and *in vivo* was performed using an IVIS Spectrum Optical Imager (Revvity).

**Figure 2:**
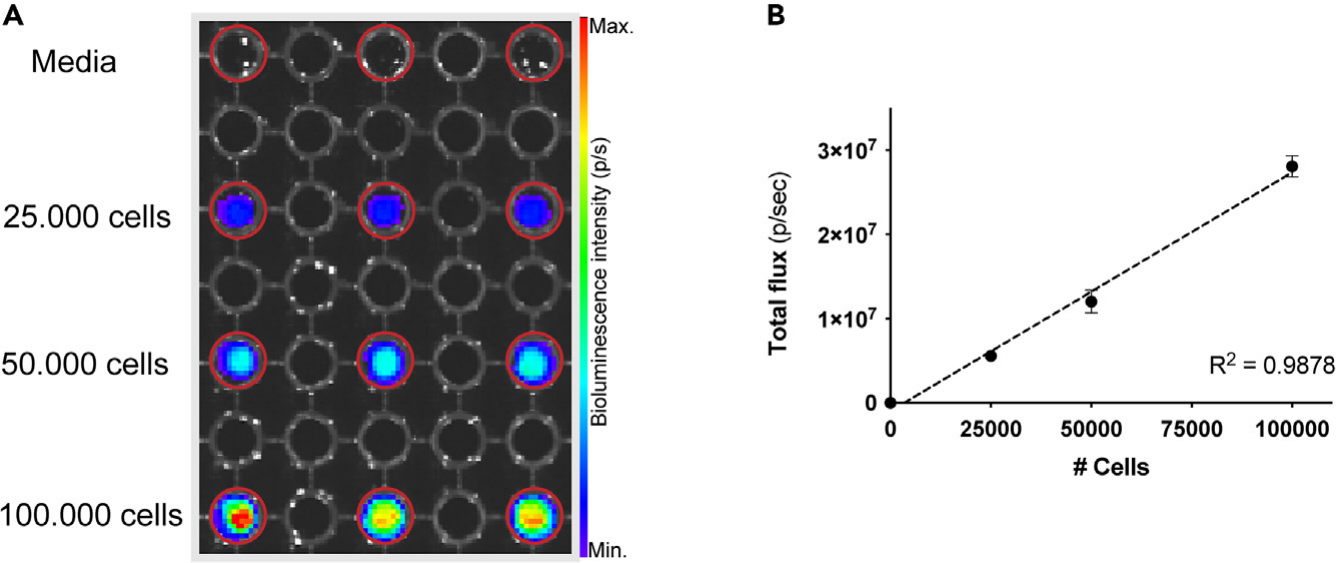
*In vitro* bioluminescence imaging and quantification of AML-PDX^LUC^ cells (A) Black walled 96-well plate containing dilution series of AML-PDX^LUC^ cells in triplicates. ROIs are indicated by red circles. (B) Regression curve of bioluminescence intensity plotted as total flux (p/sec) versus cell number. The coefficient of determination for the regression curve is 0.9878.

29. Measure the bioluminescent intensity of AML cells by optical imaging.
  a. In a black-walled, flat-bottomed 96-well plate, perform dilutions of 25.000, 50.000 and 100.000 cells in triplicates in a volume of 100 μL media per well.
  b. Leave one empty well between the wells containing cell suspension to prevent signal bleed-over from the neighboring cells.
  c. Add 10 μL of stock Luciferin 25 mg/mL to each well and mix well.
  d. Incubate for 10 min in the dark at room temperature.
  e. Remove the lid and place the plate in the imaging system.
  f. Image the cells according to the imaging systems manufacturer’s instructions.
  g. Quantify the bioluminescence intensity for each well using the software associated with your imaging system by drawing regions of interest (ROIs) around the wells containing the cells (Figure 2A).
  h. Extract the total photon count per well and plot against cell number (Figure 2B).
  i. Calculate the bioluminescent intensity per cell by dividing the total photon count by the number of cells in each well.
  j. Calculate the mean intensity per cell for each dilution.

**CRITICAL:** Bioluminescent intensity for *in vivo* experiments using the IVIS Spectrum optical imager is recommended to be approximately 200–2000 photons per second per cell. This range ensures the signal is sufficient for tissue penetration and should limit detector saturation. However, for models exhibiting an aggressive phenotype and rapid *in vivo* cell proliferation, a lower bioluminescence intensity may be sufficient. The model described in this protocol displays such characteristics and has a bioluminescence intensity of 250 photons per second per cell. AML-PDX cell engraftment in the bone marrow is detectable as early as three days post-implantation.

**Figure 3:**
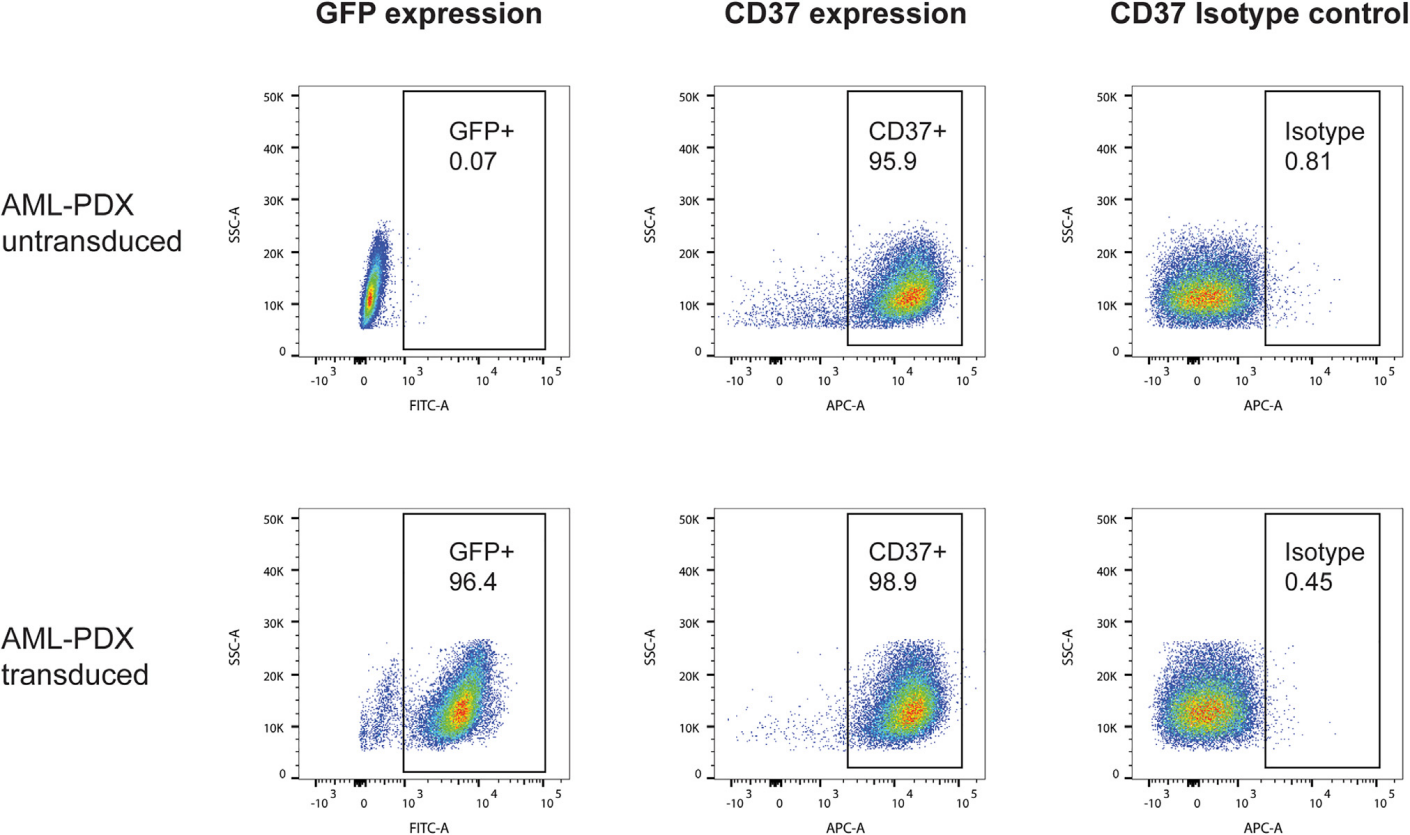
Flow cytometry plots showing the percentage of GFP positive cells, CD37 positive cells, and IgG isotype control positive cells for AML-PDX untransduced cells (upper row) and for AML-PDX successfully transduced cells (lower row)

***Note:*** When the cells have been validated for bioluminescence performance, we recommend performing a flow cytometry assay, including appropriate controls, to compare the expression of the target of interest between the non-transduced (AML-PDX) and the transduced (AML-PDX^LUC^) cells**.** This step will ensure that the target of the CAR T cell is still present after the AML-PDX^LUC^ development. See Figure 3.

**Figure 4:**
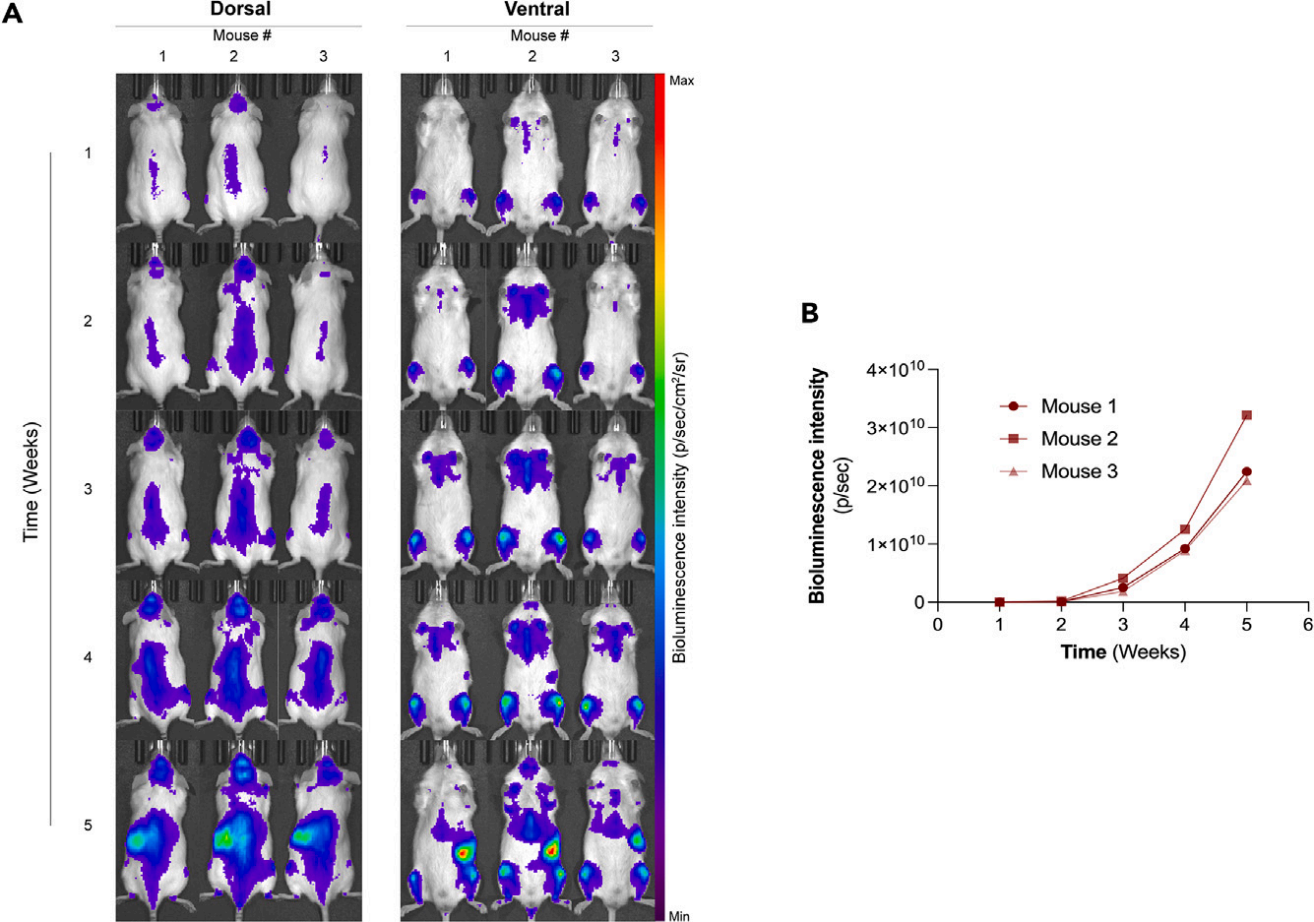
*In vivo* bioluminescence imaging and quantification of AML-PDX^LUC^ mice (A) Dorsal and ventral BLI images acquired weekly for 3 NSG mice inoculated with AML-PDX^LUC^ cells. Minimum and maximum values for bioluminescence intensity are adjusted for each time point. (B) Quantification of bioluminescence intensity over time.

30. Inject AML-PDX^LUC^ cells in 2–3 immunodeficient mice following steps 15–17.
31. Acquire weekly optical images of the mice, to create a timeline for disease progression (Figure 4A). Troubleshooting 4
  a. Weigh the mice and use the weights to calculate the amount of Luciferin necessary to image all the mice (150 mg/kg).
  b. Dissolve Luciferin in sterile saline to a concentration of 25 mg/mL.
  c. Inject mice intraperitoneally (IP) with Luciferin.
  d. Anaesthetize the mice using Isoflurane or alternative anesthesia.
  e. Acquire dorsal and ventral BLI images of the mice, 10 min after injection of Luciferin.
  f. Repeat weekly until humane or experimental endpoints are reached.
32. Quantify BLI intensity and determine to total flux for each acquired image using the Living Image software.
33. Plot the total flux data for each mouse (dorsal + ventral) to establish disease progression over time (Figure 4B).

### Utilizing AML-PDX^LUC^ to evaluate the *in vivo* efficacy of CAR T immunotherapy

**Timing: Variable (depending on the disease latency and CAR T efficacy)**

Bioluminescence imaging as a measure of AML growth is utilized to evaluate CAR T cell therapy efficacy, as well as to determine the optimal time point for treatment initiation and the appropriate frequency of administration.

34. Design your experiment by carefully selecting appropriate treatment and control groups.
35. Determine the required number of animals per group through power analysis or other suitable statistical methods.
36. Inject the mice intravenously (IV) with AML-PDX^LUC^ cells as previously described.
37. Image the mice and quantify BLI intensity according to steps 31–33.
38. Randomize the mice in groups by weight and BLI intensity.
39. Prepare CAR T cells for injection.
  a. Spin down cells by centrifuging at 350 × *g* for 5 min at RT.
  b. Resuspend in PBS.
  c. Count the cells to determine the cell concentration of each CAR T construct.
  d. Adjust the number of cells to the lowest transduction level and calculate the volume of cell suspension needed.
    ***Note:*** All CAR T cell groups must exhibit comparable levels of CAR T cell construct expression. For instance, if the lowest transduction efficiency is 80% for CD37CAR T cells, but 92% for CD33CAR T cells, the proportion of CD33CAR T cells expressing the construct should be adjusted to 80% by supplementing with Mock T cells. Mock T cells, which are transduced with an empty retroviral vector or a CAR T cell construct lacking the scFv domain, serve as controls to validate that observed differential efficacy effects of the different CAR T constructs are not due to a lower amount of CAR T cells. It is important to underline that this approach standardizes the percentage of transduced T cells but does not account for the quantity of CAR T cell molecules expressed per cell, meaning cells with the same transduction level may differ in their CAR T cell molecule density.
    ***Note:*** To account for the 80% transduction efficiency, 50 × 10^6^ cells are required to obtain 40 × 10^6^ transduced cells for injections. Assuming a starting volume of 30 mL cell suspension for each construct and the concentration and transduction efficiency of the different CAR constructs summarized in Table 1, then the calculations for each construct are as follows:
    i. Mock T cells: Calculation: 50 × 10^6^/4.8 × 10^6^. Result: 10.4 mL.
    ii. CD37CAR T cells: Calculation: 50 × 10^6^/3.4×10^6^. Result: 14.7 mL.
    iii. CD19CAR T cells: Calculation: 50 × 10^6^/1.9 × 10^6^. Result: 26.3 mL.
    iv. CD33CAR T cells (adjusted for transduction efficiency): You need 43.47 × 10^6^ cells to obtain 40 × 10^6^ transduced cells, since 40 × 10^6^/0.92 = 43.47 × 10^6^. Volume of CD33CAR T cells: 43.47×10^6^/ 3.7 × 106 = 11.75 mL. Additional Mock T cells: 50 × 10^6^ - 43.47 × 10^6^ = 6.53 × 10^6^. The additional Mock T cells volume: 6.53 × 10^6^/4.8 × 10^6^ = 1.36 mL. Total volume: 11.75 mL (CD33CAR T) + 1.36 mL (Mock T) = 13.11 mL.

**Table 1:** Overview of the number of cells and their transduction efficacy for each CAR T construct

Name	Viable cell concentration (cell/mL)	Transduction efficacy
Mock	4.8 × 10^6^	N/A
CD19	1.9 × 10^6^	80%
CD37	3.4 × 10^6^	80%
CD33	3.7 × 10^6^	92%
  e. Spin down the calculated volumes and resuspend the cells in sterile saline using the appropriate cell concentration corresponding to 100 μL per syringe, per mouse.
    **CRITICAL:** Always calculate an additional 10–20% of cells to account for the cell loss during preparation.
40. Inject CAR T cells into the mice IV.

***Note:*** BLI intensity and distribution can be utilized to determine the optimal time point for initiating treatment. Depending on the experimental objectives, treatment can begin early, when the leukemic spread is confined to bone marrow sites, or later, when blast cells are detectable in the spleen.

41. Repeat CAR T cell injection.

**Figure 5:**
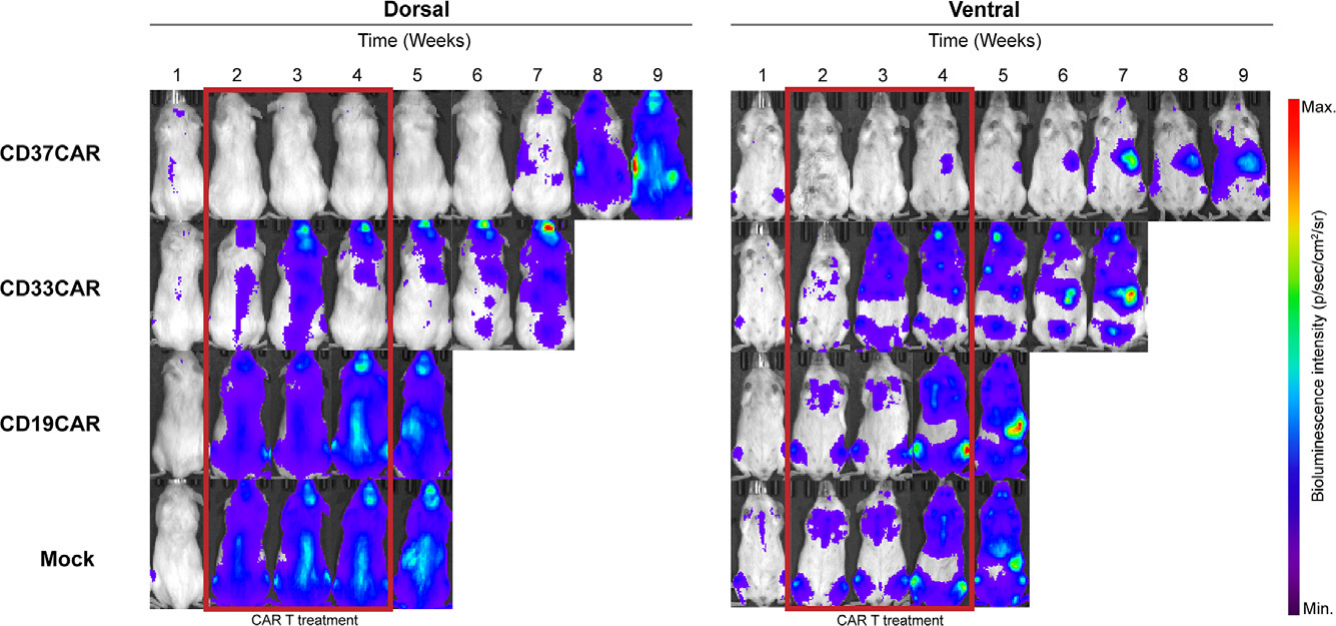
Weekly dorsal and ventral BLI imaging of AML-PDX mice representative of four different CAR T cell therapy groups: CD37CAR, CD33CAR, CD19CAR, and Mock Red rectangles indicate weeks when CAR T cells were administered. Minimum and maximum values for bioluminescence intensity are adjusted for each time point.

***Note:*** The real-time imaging results can be used to evaluate the treatment efficacy and decide the frequency of therapy as indicated in Figure 5.

## Expected outcomes

The expected outcome of this protocol is the successful development of a bioluminescent AML PDX model, which will serve as a powerful tool for evaluating the efficacy of CAR T cell therapy *in vivo*. This protocol outlines a step-by-step approach, beginning with the transduction of AML-PDX cells with a luciferase reporter gene, followed by their reinjection into immunodeficient mice to establish the bioluminescent model. The use of real-time optical imaging via bioluminescence allows for non-invasive, longitudinal monitoring of leukemic cell proliferation and dissemination throughout the host.

One of the key advantages of this method is its ability to provide dynamic insights into disease progression. By tracking bioluminescent signals, researchers can pinpoint the onset and spread of leukemia, enabling precise determination of optimal time points for initiating CAR-T cell therapy. Furthermore, the method allows for the adjustment of treatment frequency based on the real-time status of the disease burden, ensuring a more tailored therapeutic intervention (Figure 4). This approach not only enhances the relevance of preclinical testing by closely mimicking clinical scenarios but also significantly improves the ability to assess therapeutic responses and potential resistance mechanisms.

## Limitations

This protocol describes the establishment of a bioluminescent PDX model for the spatiotemporal evaluation of CAR T-cell therapy in AML. Establishing this PDX model requires immunodeficient mice to prevent the rejection of human cells, which does not fully recapitulate the human immune system. This limitation can affect the findings, particularly in understanding CAR T cells' interaction with the immune microenvironment. Further improvements, such as implanting a human immune system with the PDX material, might be superior in terms of clinical relevance.^2^^,^^3^^,^^4^

Despite the PDX model’s advantage over cell line-derived xenograft models regarding tumor heterogeneity,^5^^,^^6^^,^^7^^,^^8^^,^^9^ the bioluminescent model requires *in vitro* modification and sorting of the PDX material.^10^^,^^11^^,^^12^ Despite expanding the material *in vivo* to preserve the phenotype of the PDX,^6^^,^^12^^,^^13^ we acknowledge that this method can potentially reduce the heterogeneity of the model. Assessment of the heterogeneity of the bioluminescent model, for instance, with whole genome sequencing, might be useful to evaluate the clinical relevance of the model. Moreover, the tumor microenvironment greatly affects the functionality and trafficking of the CAR T cells. This limitation can be overcome with complementary model systems.

Another potential issue arises from excessive brightness following the transduction of PDX material, which can result in signal saturation and hinder accurate quantification of cell numbers, particularly in advanced stages of disease. This could obscure subtle changes in tumor burden. To mitigate this, the model can be adapted by selecting cells with lower bioluminescent intensity.

The process of generating and maintaining bioluminescent PDX models requires significant technical expertise, specialized equipment, and careful handling, which may not be accessible in all research settings.

## Troubleshooting

### Problem 1

Low viability of PDX cells after thawing (related to Step 1).

### Potential solution

• For long-term preservation, we recommend storing the PDX material in a nitrogen tank. The cells should be thawed at 37°C in a water bath as soon as the sample is collected from the tank or freezer. Use 20% FBS in preheated media to keep the cells stable.

In case of cell clumping, we suggest adding DNase to the medium (0.25 mg/mL), which can prevent the clumping effect caused by free-floating DNA released by lysed dead cells.

### Problem 2

There are no transduced cells.

### Potential solution

• In the case of low transduction efficacy, we recommend changing the MOI ratio, which is the number of viral particles per cell. Run a MOI titration with several ratios to determine the optimal MOI. Avoid thawing and freezing of the lentiviral particles, which reduces the transduction yield. Keep the transduction media on ice whilst not being used (Step 4).
• In the case of low transduction efficacy, one can try other protocols for cell transduction and other reagents like coating the plate used for transduction with Retronectin before adding viral particles.
• The low transduction efficacy may be due to the cells’ sensitivity to Polybrene. Polybrene generally enhances the transduction of cells, but some cells such as neurons and mesenchymal stem cells, are disturbed by it. Transduction should still be possible in the absence of Polybrene. See manufacturer’s protocol: https://www.revvity.com/no-en/asset-search/tds?part_number=CLS960003.

### Problem 3

Transduced AML-PDX cells fail to engraft (referred to Step 17).

### Potential solution

If the PDX cells do not engraft, re-evaluate the MOI used (Step 4). The handling and fitness of the PDX cells are very important. Cells could be in a bad condition after being in contact with the lentivirus. It could have been a sub-lethal number of viral particles. It can also be that the number of cells injected is too low. Some PDX models can require the injection of a larger number of cells (Step 3).

### Problem 4

The bioluminescence signal of the PDX models is saturated at advanced stages of the disease (related to Step 31).

### Potential solution

In cases where bioluminescence signal saturation occurs, it is possible to select clones with lower luminescent intensities (Steps 22 and 25). Rather than isolating populations with high GFP expression levels (Figure 1C), cells exhibiting reduced GFP fluorescence intensities can be sorted. These sorted cells should then be imaged *in vitro* to confirm that bioluminescent output falls within the optimal range of 200–2000 photons/sec/cell (Step 29). In our study, due to the aggressive nature of the disease model, a bioluminescence intensity of 250 photons/sec/cell (Figure 2B) was sufficient to detect cancer cells at early stages while avoiding signal saturation at later disease stages (Figure 4).

### Problem 5

The experiment fails to demonstrate CAR T-cell efficacy.

### Potential solution

• Re-evaluate the experimental design (Step 34). Make sure you have the appropriate number of animals per group and the correct controls (Step 35). Consider running a pilot study with a small number of mice before executing a large experiment. Increase the frequency of CAR T-cell administration or the number of cells injected (Step 39).
• Ensure sufficient transduction efficiency of your CAR T cell, preferably above 70%. If the Mock group performs unexpectedly well, consider reducing the unspecific killing by depriving the CAR T cells of IL-2, 24 h before injection.
• Test the chosen donor *in vitro* before performing *in vivo* testing to ensure good quality of the harvested PBMCs.

### Problem 6

Luciferase positive PDX model has lost the expression of the target antigen.

### Potential solution

• To avoid clonal evolution and loss of target antigen during passaging, use low-passage PDX^LUC^ material.

## Resource availability

### Lead contact

Further information and requests for resources and reagents should be directed to and will be fulfilled by the lead contact, Emmet Mc Cormack, (emmet.mc.cormack@uib.no).

### Technical contact

Technical questions on executing this protocol should be directed to and will be answered by the technical contact, Mireia Mayoral Safont, (mireia.safont@uib.no).

### Materials availability

Due to the limited amount of PDX material generated, it can be made available through a collaborative partnership.

### Data and code availability

The protocol does not include datasets.

## Acknowledgments

M.E.G. is a PhD student supported by the University of Bergen. We thank the Flow Cytometry Core, Molecular Imaging Center and Vivarium at the University of Bergen. This study was partially supported by the Research Council of Norway (NFR) AMIDE FRIPRO project (326300), Kreftforeningen IIDEA project (223171), Barnekreftforeningen projects (Ped-Hema and PERCAP) to E.M.C. and S.W., respectively, NFR KSP-2021 CellFit project (326811) to E.M.I., and NFR (337468) to S.W. This protocol was published in the scope of IMMUNO-model COST Action CA21135. We acknowledge BioRender.com as a platform used to create figures in this manuscript.

## Author contributions

M.M.S., C.L., and M.P.: conceptualization; M.M.S., C.L., and M.P.: methodology; M.M.S., C.L., M.P., and M.E.G.: validation; M.M.S., C.L., and M.P.: formal analysis; M.M.S., C.L., M.P., and M.E.G.: investigation; M.M.S., C.L., and M.P.: data curation; M.M.S., C.L., and M.P.: writing of the original draft; M.E.G., P.G., S.W., E.M.I., and E.M.C.: review and editing; M.M.S., C.L., and M.P.: figure preparation; P.G. and E.M.C.: supervision and funding acquisition.

## Declaration of interests

The CD37 CAR construct has been patented (WO2017118745A1), and S.W. and E.M.I. are listed among the inventors. E.M.C. is a co-founder, shareholder, and board member of Kinn Therapeutics AS. M.M.S. and M.P. are employees of Kinn Therapeutics AS.
